# Structure–Function Relationships of Glycoprotein Hormones and Their Subunits’ Ancestors

**DOI:** 10.3389/fendo.2015.00026

**Published:** 2015-02-26

**Authors:** Claire Cahoreau, Danièle Klett, Yves Combarnous

**Affiliations:** ^1^Physiologie de la Reproduction et des Comportements (PRC), Centre National de la Recherche Scientifique, INRA, Nouzilly, France

**Keywords:** glycoprotein hormones, luteinizing hormone, follicle-stimulating hormone, thyroid-stimulating hormone, evolution, molecular, structure–activity relationship

## Abstract

Glycoprotein hormones (GPHs) are the most complex molecules with hormonal activity. They exist only in vertebrates but the genes encoding their subunits’ ancestors are found in most vertebrate and invertebrate species although their roles are still unknown. In the present report, we review the available structural and functional data concerning GPHs and their subunits’ ancestors.

## Introduction

Glycoprotein hormones (GPHs) are the most complex molecules with hormonal activity. They include three pituitary hormones, the gonadotropins follicle-stimulating hormone (FSH; follitropin) and luteinizing hormone (LH; lutropin) as well as thyroid-stimulating hormone (TSH; thyrotropin) (1). Only in primates (2) and equidaes (3), a chorionic gonadotropin (CG) is also secreted by the placenta.

The GPHs exist only in vertebrates and appeared during evolution along with the pituitary. Nevertheless, genes coding for molecules related to GPHs subunits were identified in all vertebrates studied and in most invertebrates (4–6).

The gonadotropins FSH and LH play a central role in vertebrate reproductive function (7, 8) as they convey the integrated central information from the hypothalamic–pituitary complex toward gonads in both males and females. Indeed, internal (mainly endocrine) and external (photoperiod, congeners) information are integrated at the hypothalamus level by pulsatile gonadotropin-releasing hormone (GnRH) secretion. In mammals, GnRH is released by GnRH neurons in the portal hypothalamic–pituitary system through which it enters into the anterior pituitary. In fishes, GnRH neurons release GnRH directly into the pituitary. In all cases, GnRH stimulates the secretion of both gonadotropins FSH and LH by the anterior pituitary but their secretions are also differentially modulated by gonadal feed-backs through the action of steroid hormones and protein factors.

The TSH is also secreted by the antehypophysis but under the control of the hypothalamic neuropeptide thyrotropin releasing hormone (TRH) and is modulated by thyroid feed-back through the action of thyroxin (T4) or tri-iodo-thyronine (T3).

The placental gonadotropins (hCG in human; eCG in the mare) are secreted by trophoblast cells under no known control by any releasing hormone.

In the present paper, we will consider the structure–function relationships of GPHs and of their receptors (GPHRs) to better understand their interactions and the subsequent steps in their target cells stimulation.

## Structure of Glycoprotein Hormones and Their Ancestors

Since 1971, GPHs are known to consist of two different glycoprotein subunits, called α and β, that are non-covalently associated (9–12). This heterodimeric structure has been known for a long time to be mandatory for their respective biological functions.

The saccharide part in GPHs represents as much as 20–45% of their total mass (11, 13, 14) and has been shown to be indispensable for their *in vivo* bioactivity (15, 16). It is therefore important to get as much information as possible concerning both their polypeptide and polysaccharide portions as to decipher their respective roles.

**Figure 1:**
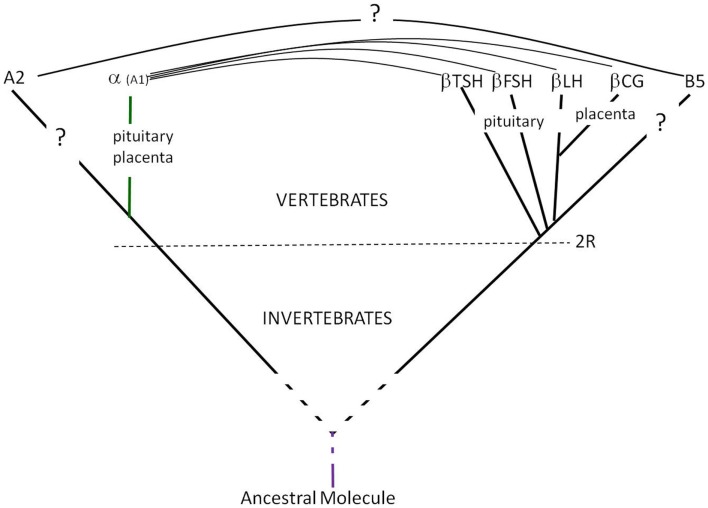
**Glycoprotein hormones’ emergence and evolution**. The GPH α- and β-subunits genes derived from GPA2 and GPB5 genes, respectively after the two rounds of full genome duplication (2R) at the origin of vertebrates (18). Both GPA2 and GPB5 are cystine-knot proteins with three loops and might derive from the same ancestral molecule.

More recently, genes encoding for proteins related to the GPH α and β subunits were found in both vertebrates and invertebrates and were named GPA2 and GPB5, respectively (4, 6, 17) and are considered as the molecular ancestors of GPH subunits (Figure 1). Recombinant GPA2 and GPB5 have been produced using plasmids encompassing the coding regions from these genes. These recombinant molecules were characterized using various immunoassays and *in vitro* bioassays. The natural GPA2 or GPB5 proteins have never been isolated but were detected in adult rat pituitaries by immunohistology and western blotting using antibodies raised against the recombinant proteins (6). The putative GPA2/GPB5 heterodimer has been described to exert thyrostimulating activity (i.e., the name thyrostimulin coined for it) (6).

### Polypeptide part

The GPHs α- and β-subunits are encoded by different genes (i.e., they do not originate from post-translational proteolytic maturation of a unique precursor like insulin subunits).

The α-subunits of all GPHs in a given species are encoded by a same and unique gene that is expressed in pituitary gonadotrope and thyrotroph cells of all vertebrates as well as in chorionic syncytiotrophoblastic cells of primates and equidaes. Therefore, the GPHs α-subunits all exhibit the same amino-acid sequence in a given species.

By contrast, the β-subunits are different and specific for each hormone. Therefore, there are at least three genes encoding β-subunits in all vertebrate species, namely FSHβ, LHβ, and TSHβ. In the human species, there are not only one but several genes encoding the hCGβ subunit.

The two subunits are thus co-translated and they non-covalently combine in the endoplasmic reticulum of gonadotrope (FSH, LH), thyrotroph (TSH), or trophoblast (CG) cells. No information is available for the time being concerning the natural GPA2 and GPB5 proteins.

#### Primary structure

All GPH subunits sequences as well as those of GPA2 and GPB5 exhibit a signal peptide at their N-terminus indicating that all these molecules are secreted glycoproteins.

**Figure 2:**
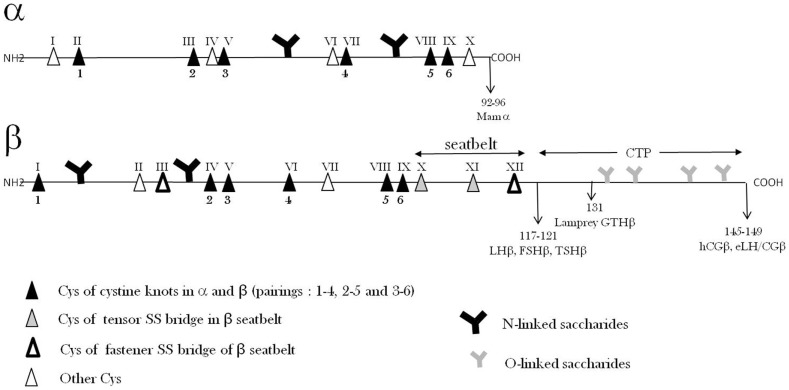
**Glycoprotein hormone α- and β-subunits’ primary structures**. The relative positions of Cys residues and N- and O-saccharide chains along the amino-acid sequences are shown.

The common α-subunits (GPA1) of mammalian GPHs after excision of their signal peptide exhibit 92 or 96 amino-acid sequences among which 10 are cysteine residues. Since no cysteine residue is in the reduced state, the α-subunits possess five disulfide bridges (Figure 2). In other species, the maturated α subunits also count approximately 90–100 amino-acid residues (19).

The amino-acid sequences of α-subunits are very well conserved among vertebrate species and in particular the positions of the 10 cysteine residues. The residues are found in groups of two or three along the sequence leaving three sequence portions without cysteines that are expected to form loops.

In the α-subunits, two potential N-glycosylation amino-acid sequences (Asn–X–Ser/Thr) are found and both of them are indeed occupied by oligosaccharide chains.

The amino-acid sequences of common α-subunits from all vertebrates exhibit a fairly high percentage of identity. This is well illustrated by the fact that it is possible to recombine these α-subunits with β-subunits from the other GPHs and from phylogenetically distant species (20, 21).

The GPA2 amino-acid sequences derived from the gene sequences in numerous species indicate that there are two potential N-glycosylation sites but one is not at the same location as in α-subunits.

The specific β-subunits (FSHβ, LHβ, TSHβ, and CGβ) polypeptide sequences exhibit around 105–150 amino-acids (19). Although different, the β-subunit amino-acid sequences exhibit large similarities which are probably largely due to their main common characteristic which is to associate with a common α-subunit. The conserved sequences in the β-subunits are important for (1) direct interaction with α and (2) similar global folding.

All β-subunits possess 12 cysteine residues which are all implicated in the six intra-chain disulfide bridges. In spite of their different sequences that determine the specificity of the different hormones, the β-subunits share a number of common features. In particular, the positions of the 12 cysteine residues in their sequences are highly conserved so that they are expected to share a common global folding (see below). The repartition of cysteines along the amino-acid sequences of β-subunits also leaves sequences without cysteines that are expected to form three loops (L1, L2, L3).

Some mammalian β-subunits (hCGβ, eCGβ, and eLHβ) possess an extension of approximately 30 amino-acid residues at their C-terminus that is called carboxy-terminal peptide (CTP). This extension has of course occurred independently in primates and equidaes through stop codon frameshift mutations leading to readthrough of the previously untranslated 3′ downstream nucleotide sequence (22, 23).

The GPB5 amino-acid sequences derived from their gene sequences show that this molecule is shorter than β-subunits by approximately 15–20 residues and misses cysteine residues III and XII present in β-subunits (Figure 2).

All GPH β-subunits possess one or two potential N-glycosylation amino-acid sequences (Asn–X–Ser/Thr), which are found in conserved positions among the different species. The GPB5 amino-acid sequences derived from the gene sequences in numerous species indicate that there is one potential N-glycosylation site but at a different location than in β-subunits.

#### Secondary structure

The circular dichroism (CD) analyses of GPHs had shown a long time ago that they exhibit a limited amount of periodic (secondary) structures: only ~4–8% α-helix and ~30% β-sheet and β-turn (24–30). From these data, the rest of the molecule was expected to be non-periodic. Interestingly, the combination of subunits was paralleled by an increase in β-structure as determined by CD (31, 32).

The three-dimensional (3D) structure of hCG determined by X-ray diffraction (33, 34) confirmed the low amount of α-helix but the proportion of β-sheet and β-turn appeared more important than expected from CD data. Indeed, a very large β-sheet involves sequences from both subunits and this structure is suspected to be important for their heterodimerization. It is likely that the increase in β-structure as determined by CD is due to the formation of this common larger β-sheet involving peptide portion from both subunits.

A possible explanation for the difference in β-structure proportion as determined by CD and X-ray diffraction is that the first one is carried out in solution whereas the second is performed in crystals. It has been shown by hydrogen isotope exchange that gonadotropins exhibit a highly mobile conformation in solution (35). It is likely that this structural dynamics observed in solution is restrained inside the crystal.

#### Tertiary structure

The folding of hCG subunits (tertiary structure) has been determined by X-ray diffraction (33, 34, 36). The determination of this 3D structure has also permitted to find out the pairings of cysteines in each of the 11 disulfide bridges (5 in α; 6 in β). An outstanding structural feature has been discovered concerning disulfide bridges: three of them in α and three of them in β form a cystine-knot with similar pairings (Figure 2) (37–39). In each cystine-knot, a disulfide bridge passes through the frame formed by two other parallel disulfide bridges joining two amino-acid sequence stretches. Such a structure, found only in a limited number of proteins including TGFβ family (TGFβ, BMPs, activin, inhibin, etc.) (39), is highly stable and forms the core of the 3D folding of the two subunits (37, 38). In the 3D structure, the three loops in α-subunits and the three loops in β-subunits can be observed that extend from the cores of each subunit.

#### Quaternary structure/heterodimerization

The quaternary structure of glycoprotein consists of heterodimerization of one α-subunit (common) with one of the specific β-subunits. In the association of the two subunits, the L1 and L3 loops of one subunit is aligned with the L2 loop of the other (33, 34). As previously stated, a large β-sheet structure formed by complementary regions from the α- and β-subunits is probably responsible for their non-covalent association.

Association–dissociation equilibrium constants of GPH subunits were found to be around 10^−7^–10^−6^ M (40, 41). These values are much higher than the GPH physiological circulating concentrations (10^−11^–10^−9^ M) and therefore the hormones should be totally dissociated and inactive at these concentrations. In fact, it has been shown a long time ago that GPH integrity *in vivo* is a kinetically regulated process (41), i.e., the equilibrium is reached very slowly giving enough time for the heterodimer to exert its action. This view has been validated later when the “seatbelt” structure was discovered. Indeed, an outstanding feature of this quaternary structure is that a sequence portion of the β-subunit, from Cys β X to Cys β XII, wraps around the α-subunit, forming a “seatbelt,” which is fastened by a disulfide bridge between Cys residues β III and XII (Figure 2) (33, 34).

It is noteworthy that the seatbelt sequence as well as the two cysteines forming the fastener in β-subunits are missing in the GPB5 sequences rendering unlikely the existence of a stable GPA2/GPB5 heterodimer. In line with this view, recombinant GPA2/GPB5 heterodimers were evidenced by SDS-PAGE and Western blotting only after chemical cross-linking (6, 42, 43) indicating that that the recombinant GPA2/GPB5 heterodimer is highly unstable.

It is tempting to postulate that GPA2/GPB5 heterodimerization is required for bioactivity as it is the case for GPH α and β subunits. The princeps paper (6) described thyrostimulating activity for the GPA2/GPB5 heterodimer and further studies led to the same (44) or other proposals for GPA2/GPB5 bioactivities in mice (45) and insects (46, 47). Nevertheless, it was not clear from these papers whether GPA2 and GPB5 were chemically cross-linked in GPA2/GPB5 heterodimers used in the bioactivity studies. Thus, it cannot be ruled out that GPA2 and/or GPB5 exert biological functions of their own (48, 49).

#### Cooperative folding

The thermodynamics of GPH subunits combination has been studied by microcalorimetry and the loss of cooperative folding was observed only at high *T*_m_, i.e., above 70°C (50). Short-term incubations (5 min) of different GPHs at temperatures between 4 and 86°C followed by sandwich ELISA to detect residual heterodimeric molecules, led to similar data (51). The conservation or restoration, of the 3D structure at fairly high temperatures was also confirmed by measuring residual bioactivity in cell-culture assays.

These data indicate that the presence of one cystine-knot in each of the two subunits together with the fastened β-subunit “seatbelt” around the α-subunit, ensure very stable tertiary quaternary structures even if it has been shown to be highly mobile by hydrogen isotope exchange of peptide protons (35).

#### Kinetics and equilibrium of seatbelt fastening

The β-subunit “seatbelt” around the α-subunit fastened by the β III–XII bridge does not influence the *K*_d_ of the subunits combination (~10^−6^ M) but considerably lowers the rate of subunits dissociation at physiological hormone concentration (~10^−11^–10^−9^ M). The two disulfide bridges in the seatbelt, β III–XII (latch) and β X–XI (tensor) appear to be involved in its opening and closing during the heterodimer αβ formation in the endoplasmic reticulum (52–54). Indeed, in the presence of a mixture of oxidized and reduced glutathione mimicking the endoplasmic reticulum redox potential, the dissociation rate of subunits is considerably accelerated. In contrast, at the much more oxidizing redox potential corresponding to that of serum, the seatbelt remains fastened and the dissociation rate of subunits is extremely slow (40).

Therefore, the hormone remains dimeric, and active, during its time of presence in the circulation even if its *K*_d_ is unfavorable (1000–100,000-fold higher than hormone circulatory concentrations).

It is important to point out that GPB5 protein sequences derived from their gene sequences lack the polypeptide portion forming the “seatbelt” as well as the two cysteine residues (III and XII) forming the “seatbelt fastener” (4, 55) in all GPH β sequences. This argues against the existence of stable secreted GPA2/GPB5 heterodimers (38, 48).

### Polysaccharide part

The mammalian GPHs contain from 15 to 45% saccharide in mass. Polysaccharide chains are only N-linked in most of these hormones (two in α and one or two in β; Figure 2) (13). However, a few of them (hCG, eCG, and eLH) also possess O-linked saccharides on their CTP extension in their β-subunits (13, 56) (Figure 2). These sugar moieties confer high solubility, increase apparent molecular mass in SDS-PAGE, and charge polymorphism of the GPHs. These physico-chemical properties also convey numerous important functional properties to them. It is therefore important to consider them here.

#### N-linked saccharide chains

The positions of N-linked saccharide chains are determined primarily by the presence of potential N-glycosylation amino-acid sequences (Asn–X–Ser/Thr). Saccharide chains are transferred “en bloc” in the endoplasmic reticulum to these Asn residues in the polypeptide chains during the course of their translation. At this step, the N-linked saccharide chains are of the immature type, i.e., high-mannose and glycosylated. Interestingly, these high-mannose type N-glycans have been described to possess chaperone-like function during protein folding in endoplasmic reticulum (57) and accordingly they facilitate correct disulfide bond pairing (58). Correct folding is assessed by a quality control system consisting of chaperone proteins such as calnexin, BiP, and/or Grp94 (59). This quality control also involves the removal of the three glucose residues from these chains before the transfer to the Golgi apparatus.

Maturation of the N-linked chains then occurs in the Golgi apparatus. It consists in partial mannose removal by two mannosidases, addition of GlcNAc, Gal, and sialic acid residues by specific enzymes. The enzyme assortment in different cell types can differ so that N-saccharide chains can differ from one cell type to the other. This is particularly obvious for equine LH and equine CG that are encoded by the same α- and β-subunit genes.

These hormones are synthesized in the pituitary and placenta respectively and although they share the same polypeptide chain sequences, they exhibit largely differing sugar moieties (56). The N-saccharide chains are of complex or hybrid types and possess one to four antennas which are either completed up to a terminal sialic acid residue or not.

Concerning GPA2 and GPB5, only recombinant molecules and no natural ones have been available for structural studies. Therefore, it is possible to spot potential N-glycosylation sites in their sequences but the presence or absence of saccharide chains at these sites can only be checked in proteins synthesized in heterologous systems (CHO, HEK, Sf9 cells). The analysis of GPA2 sequences shows the presence of two potential glycosylation sites at the same locations in vertebrates and the urochordate *Ciona intestinalis* and of only one at a completely different location in the cephalochordate *Branchiostoma* and in protostomes. This observation reinforces the view that among chordates, urochordates are phylogenetically closer to vertebrates than cephalochordates (60). Concerning GPB5, there is one potential N-glycosylation site in all the analyzed deuterostomes (mammal vertebrates, *Ciona* protochordate, *Strongylocentrotus purpuratus* echinoderm) but at different locations along their respective amino-acid sequences. By contrast, none was found in the protostome sequences.

#### O-linked saccharide chains

O-linked saccharides are found in GPH heterodimers possessing a CTP at the C-terminus of their β-subunits (hCG, eCG, and eLH). Four O-linked saccharides are found in the hCG CTP whereas as much as twelve of them can be found in eLH and eCG CTPs. The numerous O-saccharides found in the CTP are thought to impede compact folding of the polypeptide chain and to keep it extended (61).

The α-subunit is synthesized in excess relative to the β-subunits in the pituitary, and interestingly, it has been found that the excess free α molecule partly undergoes an O-glycosylation at its Thr43 residue (51, 62–65). For hCG, this O-glycosylation at Thrα43 is a late event in the secretory pathway that occurs in the Golgi apparatus whereas αβ combination occurs earlier in the endoplasmic reticulum and therefore O-glycosylation of free α plays no role in the heterodimer assembly.

Interestingly, the free α molecule has been found to play paracrine roles in the pituitary and the placenta (63, 66–70).

The molecular compositions of O-linked saccharides are variable (71). Some of them are composed of only three saccharide units whereas others are extremely long with poly-lactosamine extensions. These long extensions in addition to the unfolding effect of multiple O-saccharide sites on the polypeptide chain are responsible for the high apparent molecular weights of the CTPs in hCG and eCG.

#### Isoforms

The differences in their number of antennas and in their completion render N-saccharide chains extremely heterogeneous in terms of mass and charge. Probably hundreds of hFSH isoforms might exist due to all possible variants of their four N-saccharide chains (72). The number of potential isoforms is even higher in the case of GPHs with both N- and O-saccharide chains such as hCG, eCG, and eLH but less variability was observed in hCG N-linked carbohydrates than in those in hFSH.

Due to this mass and charge heterogeneity of their saccharide chains, GPHs exhibit an important polymorphism in electrophoresis or chromatofocusing. Such a large polymorphism makes the physico-chemical characterization of GPH preparations very difficult. This is true for natural as well as for recombinant hormones which must be produced in eukaryotic cells in order to be glycosylated.

Polymorphism due to size and charge heterogeneity of their saccharide moieties has also been found to vary as a function of physiological situations (menstrual cycle, gender, age) (73, 74) and occasionally as a consequence of pathological situations.

In the case of TSH, it has been recently reported in the mouse that the hormone produced by the pituitary pars-distalis (PD-TSH; ~35 kD) essentially bears biantennary and sulfated N-linked carbohydrate chains whereas that produced by the pituitary pars-tuberalis (PT-TSH; ~40 kD) bears sialylated bi-, tri-, and tetra-antennary carbohydrate chains (75). It is reported by these authors that only PD-TSH stimulates thyroid hormones secretion whereas PT-TSH acts only on the hypothalamus to regulate seasonal physiology and behavior. The different glycosylations of these two tissue-specific TSH isoforms are thus responsible for their differing functional properties *in vivo*. This difference is not attributable to differing TSH receptors (TSHRs) in the thyroid and hypothalamus but to different affinities toward IgG and albumin leading to differing spatial distributions of the two isoforms (75).

### Structure and immune properties

Immunologic properties of GPHs have been used for a long time in order to set up immunoassays to measure their concentrations in blood in normal physiological conditions as well as in pathological situations. Also, GPHs can promote the production of antibodies that can adversely affect their function. It is thus of interest to consider these properties for a better understanding of their physiological roles and in view of a better control of their activity in clinical situations where they are injected to patients.

For the setting-up of immunoassays, it is important to raise antibodies that can distinguish the different GPHs. Therefore, these antibodies are most often directed against the specific β-subunits. Nevertheless, it is often more efficient to raise antibodies against the heterodimer and to select either those that are specific of the conformation taken by the β-subunits when associated with the common α-subunit or those that recognize epitopes contributed by both subunits in the heterodimer. The free α-subunit is not very immunogenic but a number of antibodies against the heterodimers recognize epitopes in the associated α-subunit.

Monoclonal antibodies against each subunit are particularly valuable for the setting-up of sandwich ELISAs that specifically detect the heterodimers and differentiate them from free subunits.

## Half-Life and Elimination of Gonadotropins from Circulation

The contribution of carbohydrate chains to GPHs half-lives has been known for a long time (76–78). Their size and charge both contribute to their maintenance in the circulation. There are two main routes of elimination of gonadotropins from blood: liver capture (78) and more prominently kidney glomerular filtration (79–81).

For bulky plasma glycoproteins, half-life is essentially determined by their hepatic capture (76). Indeed, the -Gal-NANA end of N-saccharide branches can more or less rapidly be desialylated so that the Gal residue becomes exposed and recognized by the hepatic Gal receptor (82). This leads to removal of the glycoproteins from circulation. Experiments in which hepatic circulation in piglets is partly bypassed from the hepatic portal vein to the vena cava do not show any significant increase in LH half-life (79). It is therefore likely that the liver is not the most important route of GPH elimination from circulation. The apparent molecular weights of GPHs are far below the glomerular filtration limit (~60 kD) and therefore they are readily eliminated through urine (except eCG). The high concentrations of bioactive hCG in pregnant women’s urine and of bioactive LH and FSH in post-menopausal women’s urine clearly demonstrate that these hormones are readily filtrated through renal glomerulus without major alteration of their structure. Accordingly, the half-lives are extremely short (5–30 min) for GPHs without CTP such as hFSH and hLH.

Also in keeping with this, a mutated recombinant hFSH with four additional N-linked glycan chains was found to exhibit a longer half-life and consequently a higher *in vivo* activity (83). Nevertheless, liver mannose receptors appear to be involved in the removal from circulation of glycoprotein with glycans terminated by β1,4-linked GalNAc-4-SO4 such as in LH mainly (84, 85).

Placental GPHs possessing a CTP with long O-carbohydrates exhibit longer half-lives (1.5–2.5 days) than pituitary GPHs without CTP (5–30 min). Indeed, the hydrophilic long saccharide chains borne by the CTP are very bulky and negatively charged because of their terminal sialic acid residues. These properties lead to lowered glomerular filtration because of the size and negative charges of glomerular pores. This explains why eCG which possesses the greatest and most acidic saccharide chains on its CTP is not found in urine in contrast to LH, FSH, and even hCG as mentioned above.

**Figure 3:**
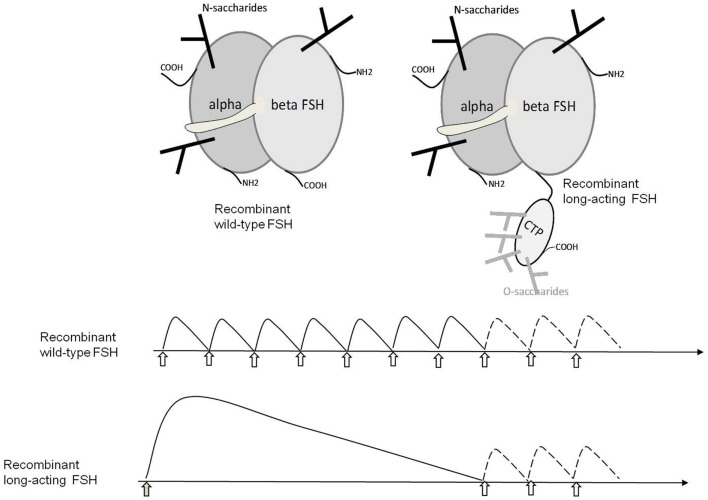
**Structure of wild-type and long-acting hFSHs and their therapeutic use**. The upper panel schematically shows the structure of wild-type hFSH and hFSH fused with the hCGβ CTP that confers extended half-life to the heterodimer in the circulation. The lower panel shows the usage of wt-hFSH injected daily for 1 week and then optionally for 1–3 days more depending of follicular development. LA-FSH is injected once and the treatment is optionally continued with one to three daily injections of wt-FSH if required.

This unique property of βCTP has been exploited by fusing hCG βCTP to the hFSHβ subunit sequence in recombinant hormones in order to increase its half-life in circulation (86–88) (Figure 3). In further works, βCTP was used as a tether between subunits to produce various single-chain GPHs (51, 89–93).

## Molecular Mechanisms of Action of Glycoprotein Hormones

The very first step in GPH action is their binding to specific receptors (GPHRs) at the plasma membrane level of gonadal cells. GPHRs are seven transmembrane domain (7-TMD) receptors with a very large extracellular domain (ECD) containing numerous leucine-rich repeats (LRR) (94) with a horseshoe shape that accommodate GPH binding.

Following high-affinity and specific binding with the receptor’s ECD, an interaction with the 7-TMD must occur to promote a transconformation that is detected by the intracellular partners, essentially the heterotrimeric G-protein Gs but also, in certain situations, other pathways (95).

### Binding to specific receptors

In spite of their respective large similarities, GPHs specifically interact with their cognate GPHRs. This is true at least when considering hormones and receptors from the same species. Indeed, eLH and eCG are known to exhibit FSH activity in addition to their LH activity in all species except their own (horse) and chicken. Moreover, hCG exhibits low but significant TSHR binding activity in addition to its strong LHR binding activity even in its own species (human).

It has been known for a long time now that both the GPH common α- and the specific β-subunits participate in receptor binding (95, 96). It appears from X-ray diffraction studies of the FSH–FSH ECD complex that the β-subunit’s seatbelt and the two neighboring regions in the α-subunit form the hormone surface that interacts with the receptor’s ECD (36, 97, 98). The involvement of the common α-subunit in receptor binding suggests an important similarity in the interaction mechanisms. From comparative data, we were the first to propose a long time ago the “negative specificity” model. In this model, the α-subunit contribution to binding is only high-affinity whereas that of the β-subunit’s seatbelt is to control specificity by inhibiting hormone binding to the “wrong” receptors (99, 100). It is interesting to point out that GPB5 that lacks this seatbelt region not only would lead to unstable GPA2/GPB5 heterodimers but also would lack this specificity mechanism leading to their potential binding to all GPHRs. Since TSHR is the receptor with the lowest specificity as it also binds hCG (101, 102), this may explain why GPA2/GPB5 binds only to it and not to FSHR and LHR.

The crystal structure of the FSHR ECD in complex with FSH (36, 95, 103) indicates that FSH establishes contacts with some β-strands of the concave inner surface of the leucine-rich repeat domain (LRRD) of the ECD (94). This paper also report dimerization of the FSH/FSHR ECD complex in agreement with previous data showing that recombinant GPHRs functionally defective either in their ECD or 7-TMD can complement and transduce the hormonal signal when co-expressed in the same cells (104, 105). More recent data suggest a trimerization of FSHR upon FSH binding (106) and an activation mechanism involving a FSH transconformation allowing its direct interaction with the FSHR sulfated Tyr residue at position 335 (95).

In the TSHR, the hinge region does not only serve for stabilizing the receptor ECD LRRD but it also participates in TSH binding (107).

The position of certain N-saccharide chains can affect specificity of binding. Indeed, it has been shown that eCG and eLH do exhibit FSH activity in all species tested so far but not in their species of origin (horse) and also very weakly in chicken (108). As mentioned above, the presence of an extra saccharide chain at the same position (Asn 268) in the FSH receptors ECDs from these two species might explain why LH specificity of eLH and eCG only exists in these species.

It has been reported that the GPA2/GPB5 heterodimer binds to the TSHR in mammals (6, 42, 109) but the receptors for GPA2/GPB5 or GPA2 and GPB5 independently in invertebrates are obviously not TSHR but are suspected to be GPHR ancestors and thus to belong to the GPCR family exhibiting a LRRD. In *Drosophila melanogaster*, the DLGR1 GPCR with LRRD has been reported to bind human GPA2/GPB5 (43). The LGR1 receptor gene has also been identified in the mosquito *Aedes aegypti* where this receptor has been proposed to regulate ion-transport across the hindgut (47). In *Caenorhabditis elegans*, only one GPCR gene with LRRD has been identified (*fsh-r1*) that exhibits the highest similarity with FSHR among mammalian GPHR and plays a role in innate immune response (110). In this species, genes apparented to GPA2 (*flr-2*) and GPB5 (*flr-5*) were identified and might regulate FSH-R1 in the neural control of intestinal functions (49), maybe including intestinal ion-transport and/or intestinal immune defense.

### Transmembrane signal transduction

The transmembrane (TM) signaling by GPHRs is due to the transconformation of their 7-TMD upon hormone binding to their ECD and subsequent interaction with the extracellular loops and/or extracellular amino-acids of their 7-helical TM sequences.

Precious structural information has been gained from the crystallographic studies of hormone–receptor ECD complexes mentioned above. No such information is available concerning the whole receptor and thus concerning ECD/7-TMD interaction upon GPH binding.

It had been shown a long time ago that LH is able to activate its receptor deprived of its ECD but with a low *K*_d_ (10^−7^ M instead of 10^−10^ M approximately) (111). This result suggests that the hormone ligand itself can directly contact the 7-TMD albeit with low affinity and promote its activating transconformation. The ECD might thus “catch” the hormone at very low physiological concentration and greatly favor its interaction with the 7-TMD so that transconformation of the latter can occur. In this first model, the hormone would thus be sandwiched between the ECD and the 7-TMD.

Determination of the 3D structure of GPCR 7-TMD has succeeded in only a limited number of them giving a static view yet they are expected to be highly dynamic and to assume different conformational states. These different conformations are functionally important as they are stabilized through the binding of the receptor with agonists or antagonists, with another receptor (dimerization) or with downstream partners (proteins G or others). These trans-conformations between inactive and active states affect mainly the relative orientations and distance of the seven TM sequences that can be detected intracellularly by the downstream partners (112). Repulsive separation of TM3 and TM6 in the TSHR 7-TMD is linked to receptor constitutive activation (113).

Glycoprotein hormone receptors are GPCRs and as such convey their signaling information mainly through interaction with heterotrimeric G-proteins mainly Gs but also Gi or Gq. Upon stimulation by their cognate GPH, GPHRs interact with the α-subunit in Gs protein and promote the exchange of GDP for GTP in this α-subunit. The α_s_-GTP subunit then separates from the βγ subunits complex and interacts with the adenylate cyclase to increase its activity and thus intracellular cyclic AMP concentration (114). This very general mechanism will not be detailed here.

### Intracellular signaling downstream to GPHRs

The main partner GPHRs is the heterotrimeric Gs protein that stimulates membrane adenylate cyclase activity and consecutively leads to intracellular increase in cyclic AMP and thus to protein kinase A (PKA) stimulation. Specific phosphorylation of numerous proteins on threonine and/or serine residues is catalyzed by PKA. Among these proteins, there are transcription factors and various metabolic enzymes and structure proteins so that the biological responses to GPHs are genomic and/or metabolic and/or morphological. Thorough description of these cellular responses would be interesting but outside the scope of the present chapter. We focus here on such issues only for examples related to the structure of GPHs or GPHRs.

After their phosphorylation by GRK, GPHRs recruit arrestin that promotes desensitization of the Gs pathway. In addition, arrestin acts as a scaffolding protein and recruits MAPKKK (Raf), MAPKK (MEK), and MAPK (ERK) and stimulates this pathway. It is thus interesting to note that arrestin do not act solely for arresting Gs signaling but also to initiate another signaling pathway (115). Therefore, arrestin is not only an interruptor but a commutator in signaling pathways downstream of GPHRs stimulation.

### GPHR desensitization and internalization

Upon stimulation of GPHR by their cognate GPH, activated α_s_-GTP of the Gs protein separates from the βγ subunits complex. This complex recruits a GRK (GPCR kinase) that can in turn phosphorylate the activated GPHR at one or several specific locations in its intracellular sequences. These phosphorylated residues are target sites for arrestin that then interferes with the receptor–Gs protein interaction and thus desensitizes this pathway. In addition, as indicated before, arrestin also acts as a scaffolding protein that recruits MAPKKK, MAPKK, and MAPK thus initiating the stimulation of this pathway. Downstream the LH-induced LHR activation, it is the arrestin-3 isoform that is involved in the MAPK cascade activation in MA-10 cells (116).

Upon further hormone stimulation, receptor internalization occurs in addition to desensitization. This step is promoted by the arrestin-induced clathrin-coated pits that engulf GPHRs intracellularly into the endosomes. In the case of the TSHR, it is the arrestin-2 isoform that promotes internalization through this pathway (117). The fate of the internalized receptors is either degradation or recycling back to the plasma membrane in various proportions. The monomeric G-proteins Rab are responsible for this trafficking by promoting vesicle budding and fusion. These Rab proteins are GTPases like the Gs α-subunit: they are activated by exchanging GDP for GTP under the action of a guanosine nucleotide exchange factor (GEF) and they are inactivated by hydrolyzing this GTP back to GDP.

Their GTPase activity permits their auto-inactivation which is enhanced by GTPase activating proteins (GAPs). Among the 60 Rabs known so far, Rab5a which is located to early endosome, mediates GPCR internalization. Indeed, Rab5a facilitates LHR internalization but it also favors its degradation and inhibits its recycling (118).

## Physiopathological Conditions and Clinical Consequences

The most numerous diseases involving gonadotropins are due to diminished (congenital hypogonadotropic hypogonadism) or excessive (adenomas) levels of secretion. The first are due to fetal defect in GnRH neuron migration, or a defect of pituitary development or from a functional defect of the hypothalamic–pituitary axis between GnRH neurons and gonadotrope cells (Kallmann syndrome). The latter arises following the development of LH-, FSH-, or TSH-secreting pituitary adenomas (119). The study of these conditions is beyond the scope of the present paper as they are not related to the structure of gonadotropins or of their receptors.

Natural mutations in GPH genes and in their receptors’ genes are either activating (gain of function) or inhibiting (loss of function). The former are expressed as a dominant trait, thus in the heterozygous state, whereas the latter are only expressed when biallelic. In addition, some mutations affect internalization and/or degradation of GPHRs.

### Pathological conditions due to hormone structure modifications

Pathological conditions due to mutations in gonadotropin or thyrotropin gene coding sequences and hence to modifications in their polypeptide structure are fairly rare. However, some pathological conditions are due to or are related with modifications in the saccharide side chains.

For example, trophoblastic cells from trisomy 21 pregnancy produce hyper-glycosylated forms of hCG with low biological activity (120). Detection of hCG variants has also been shown to be related to various malignancies in human (121).

Abnormal stimulation of TSHR by hCG during pregnancy has also been described. A small proportion of these patients have clinical hyperthyroidism, termed gestational thyrotoxicosis (102). They either secrete a variant of hCG with increased thyroid-stimulating activity or their TSHR has increased affinity for hCG. A unique family with recurrent gestational hyperthyroidism associated with hyperemesis gravidarum was found to have a mutation in the ECD of the TSHR that made it responsive to normal levels of hCG (122).

While mutations of gonadotropin and TSH genes in human are exceedingly rare, genetic alterations of their respective receptors are more frequent. These mutations can lead either to constitutive activation of the receptors or to their inactivation, i.e., their inability to respond to their cognate hormone for various reasons.

### Pathological conditions due to FSHR

#### Inactivating mutations

Inactivating mutations of FSHR gene, in women, are generally associated with primary ovarian insufficiency. Numerous inactivating FSHR mutations have been described such as Ala189Val in the FSHR ECD leading to hypergonadotropic hypogonadism with no or weak response to FSH (123, 124). A Pro519Thr mutation in the second intracellular loop of the 7-TMD was associated to elevated serum FSH concentration, low estrogen and inhibin concentrations, and hypoplastic uterus and ovaries. The inactivity of the receptor was found not to be due to defect in its signal transduction ability but to its intracellular trapping, and therefore to its absence at the cell surface. In patients with such total loss of FSHR function, there is no passage from primary to secondary follicles (125) and this block of course causes infertility.

#### Activating mutations

In contrast to the numerous inactivating mutations in FSHR, only one example of activating mutation has been described in human: Asp567Gly in the third intracytoplasmic loop (126). The D550G mutation in the FSHR 7-TMD has been recently shown to uncouple the link between internalization and degradation of hFSH. It is therefore expected to be more efficiently recycled.

#### Extragonadal expression

In addition to FSHR structure modifications due to mutations, diverse pathologies are a consequence of its anomalous extragonadal expression. FSHR is normally expressed in extragonadal reproductive tissues such as placenta (127) but also in the endothelial cells associated with a diverse range of solid tumors (128). Genitourinary malignancies were strongly represented (prostate adenocarcinomas, urothelial carcinomas, renal cell carcinomas, and seminomas). The ubiquitous nature of FSHR in tumor blood neovasculature suggests a biological role in human solid tumors possibly through induction of vascular endothelial growth factor (VEGF) in granulosa cells. FSHR has also been shown to be expressed in normal prostate tissue but at significantly lower levels than in prostate cancer.

The presence of the Ser680 FSHR isoform in Taiwanese women was found to be associated to a lower occurrence of endometriosis (129). This suggests the presence of functional FSHR in invading endometrial cells.

### Pathological conditions due to LHR structure modifications

Activating and inactivating mutations in LHR with very different phenotypic effects have been identified. Inactivating mutations in the LHR are responsible for male pseudohermaphroditism or Leydig cell hypoplasia in individuals with 46 XY karyotypes, characterized by a predominantly female phenotype. Activating mutations in the LHR are responsible for precocious puberty due to its constitutive activity in the absence of hormone.

#### Inactivating mutations

Inactivating mutations of LHR gene most often affect XX individuals whose families also include cases of male pseudohermaphroditism. Clinically, these women suffer from primary amenorrhea but with normal development of breast and pubic hair.

Naturally occurring LHR mutant without the polypeptide sequence encoded by exon 8 causes Leydig cell hypoplasia due to the loss of hormone-binding ability. The LHR mutant lacking exon 9 was found not to be addressed to the plasma membrane thus also leading to insensitivity to the hormone. Recently, a novel cryptic exon (exon 6A) was found in LHR gene (130) that leads to the synthesis of an incomplete mRNA variant encompassing exons 1–6–6A (6A terminal variant). A mutation in exon 6A (A557C) leads to an overexpression of this shortened mRNA as well as that of an mRNA including exons 1–6–6A–7–11 (6A internal variant) in Leydig cells leading to an insufficient amount of full-length mature LHR at the cell surface. Like inactivating mutations in the LHR coding sequence, the overexpression of LHR mRNA including the 6A exon is thus responsible for male pseudohermaphroditism or Leydig cell hypoplasia (130). These three examples among many others indicate that the insensitivity of LHR to its cognate hormones LH and HCG may have very different causes (binding deficiency, intracellular trapping, or transcription inhibition).

#### Activating mutations

Activating mutations in the LHR gene are one of the most common mutations found in the GTHR genes. These mutations promote precocious puberty in boys but no obvious phenotype in females (131).

There are no reports of naturally occurring activating mutations in the ECD although, engineered mutations of a serine residue in the hinge region of LHR result in constitutive activity of receptors expressed in recipient cells (132). One of the most common activating mutations is the missense mutation D578G in TM6 of the TMD. Another mutation at the same position (D578H) was found to be highly activating. To date, there is no report of women with a D578H mutation; this mutation has appeared only as a somatic mutation restricted to Leydig cell tumors in boys ranging in age from 5 to 8 years, suggesting that this particular mutation is incompatible with germ-line transmission (131).

Another constitutively activating mutation in the TM3 of the hLHR (L457R) has been identified in only one boy with gonadotropin-independent precocious puberty (133). Interestingly, the amino-acid in this location forms a salt bridge with the amino-acid in position 578 that is also prone to activating mutations as described above. The L457R mutation in LHR has also been found to diminish lysosomal degradation of the receptor and this could also contribute to its constitutive activity by prolonging the duration of signaling (134).

The N312S mutation of LHR does not lead to any functional effect but has been shown to be moderately but significantly related to increased breast cancer (135). The reason of this link is unknown.

### Pathological conditions due to TSHR

#### Activating mutations

Activating mutations of the TSHR are rare. Nevertheless, an heterozygous substitution in exon 10 (Ile568Thr) leads to neonatal thyrotoxicosis without anti-TSHR antibodies production (136). Likewise, a Leu665Phe mutation in TSHR TM helix 7 leads to non-autoimmune hyperthyroidosis (137).

#### Inactivating mutations

Inactivating mutations in human TSHR also exist in a few occurrences. For example, the Gln489His mutation in the first extracellular loop, leads to hypothyroidism (138).

#### TSHR autoantibodies

Graves’ disease (elevated thyroid hormone levels and low to undetectable TSH) is a leading cause of hyperthyroidism worldwide. It arises from the action of TSHR stimulating autoantibodies. TSHR autoantibodies are either stimulating (cAMP/PKA/CREB and/or AKT/mTOR/S6K signaling cascades) or inhibiting TSH effects, or neutral autoantibodies that induce thyroid cell apoptosis via reactive oxygen species (ROS) generation.

In contrast to the gonadotropin receptors FSHR and LHR, TSHR ECD is maturated by proteolysis and is connected to the TM domain through a disulfide bridge (139). The unshed TSHRs or disulfide cleaved soluble ECD forms and/or TM forms might be much more immunogenic than the functional maturated receptors with SS-bridged subunits.

#### GPA2/GPB5 binding

Since GPA2/GPB5 heterodimer has been proposed to bind to TSHR in vertebrates, it is interesting to point out that GPB5^−/−^ mutant mice exhibit transient hypothyroxinemia (109) whereas mice overexpressing GPB5 show a resistance to diet-induced obesity (45). The involvement of defects in GPA2, GPB5, or TSHR should thus eventually be taken in consideration in thyroid axis pathologies.

## Conclusion and Perspectives

Because of their implication in important physiological functions and their structural complexity, GPHs and their receptors remain a difficult and active field of investigation. The complexity of their structure makes the pharmaceutical production and control of recombinant hormones a heavy task. For example, hFSH with a fused hCGβ CTP (Figure 3) which is now on the market (corifollitropin; Elonva^®^) in an increasing number of countries clearly exhibits a longer half-life that permits to diminish the number of injections to the patients. Nevertheless, there is a possible risk of ovarian hyperstimulation in some of them.

Since GPCRs are the main targets of pharmaceuticals, it can be envisioned that synthetic small-molecular weight drugs will be, sooner or later, available to finely tune the activation of LHR and FSHR for fertility treatments. A number of such molecules have already been synthesized and tested (140–144) and structure modeling (145, 146) is expected to be particularly helpful in the molecular design of new drugs of this type.

Concerning their ancestors, GPA2 and GPB5, their putative heterodimerization is still a matter of debate as well as their biological activities either on their own or after combination.

## Conflict of Interest Statement

The authors declare that the research was conducted in the absence of any commercial or financial relationships that could be construed as a potential conflict of interest.
